# Longitudinal Incidence of Kidney/Urinary Stones in Individuals With Severe Motor and Intellectual Disabilities

**DOI:** 10.7759/cureus.69071

**Published:** 2024-09-10

**Authors:** Kazuhiko Hashimoto, Shimpei Baba, Noriko Sumitomo, Eri Takeshita, Yuko Shimizu-Motohashi, Takashi Saito, Eiji Nakagawa, Masayuki Sasaki, Hirofumi Komaki

**Affiliations:** 1 Department of Child Neurology, National Center Hospital, National Center of Neurology and Psychiatry, Tokyo, JPN; 2 Department of Epileptology, National Center Hospital, National Center of Neurology and Psychiatry, Tokyo, JPN; 3 Translational Medical Center, National Center of Neurology and Psychiatry, Tokyo, JPN

**Keywords:** cumulative incidence rate, health burden, kidney/urinary stones, prognosis, severe motor and intellectual disabilities

## Abstract

Objective and rationale: To investigate the longitudinal incidence of kidney/urinary stones in patients with severe motor and intellectual disabilities and explore health burden events in patients with stone formation.

Methods: This was a retrospective, observational study. We identified patients with severe motor and intellectual disabilities who had the following: 1) admission to our hospital wards for >10 years; 2) two or more assessments for stone formation by ultrasonography or computed tomography; and 3) absence of kidney/urinary stones in the first imaging study. The Kaplan-Meier method was used to analyze the cumulative incidence of kidney/urinary stones. Recurrent urinary tract infections, hydronephrosis, renal dysfunction, and death were identified as health burdens.

Results: Among the 41 patients (19 men, 22 women; median age, 28 years; range, 8-50 years), stone formation was detected in 11 (27%) patients during the observation period. The cumulative incidence rate of stone formation was 9.8% (95% confidence interval, 3.8-23.9) and 18.7% (95% confidence interval, 9.2-35.7) at five and 10 years, respectively. Death was frequently observed in patients with stone formation; six (55%) of the 11 patients with stone formation died during the follow-up period; two (15%) died among the other 30 patients without stone formation. However, only one patient with stone formation died in a renal event; the causal relationship between the stone formation and the deaths was not clarified.

Conclusion: The longitudinal incidence of kidney/urinary stones was higher in patients with severe motor and intellectual disabilities than in the general population. Considering the difficulty of patients with severe motor and intellectual disabilities in conveying their symptoms, regular assessment of the kidney using abdominal imaging may be recommended.

## Introduction

Severe motor and intellectual disabilities (SMID) refer to profound intellectual disability (IQ < 20) and the inability to move independently [1,2]. Individuals with SMID usually have chronic and multiple physical problems, including epilepsy, sensory impairments (e.g., visual impairments), abnormal muscle tone (e.g., spasticity), skeletal deformations (e.g., scoliosis and hip dislocations), bladder and rectal disturbances, and gastrointestinal dysfunction (e.g., constipation, gastroesophageal reflux, and esophageal hiatal hernia), in addition to profound neuromotor dysfunctions [3,4]. SMID may be confused with cerebral palsy (CP); however, CP does not always equal severe physical and/or intellectual disability [5]. In contrast, SMID can include CP and postnatal brain damage, such as hypoxic-ischemic brain injury, traumatic brain injury, central nervous system infection, and progressive brain diseases.

Owing to recent advancements in medical care, a growing number of children with SMID become adults with increasing burdens at global levels [6]. Several studies have reported that the life expectancies of children with severe disabilities have improved in developed countries [7-9]. A recent study from Australia showed that more than 80% of individuals with CP have a life expectancy longer than 58 years [8]. Despite the marginal statistical deference, the mortality risk improved from the 1960s birth cohort to the 2010s [8]. Another study from Korea described that, between 2004 and 2017, the life expectancy for individuals with disabilities increased by 9.1 years in men and 8.3 years in women, whereas for non-disabled, it increased by 5.5 years in men and 4.6 years in women, respectively [9]. Life expectancy decreased according to the severity of the impairment, but even in individuals with the most severe disability, life expectancy increased from 40.7 to 49.7 years [9]. However, clinicians caring for patients with SMID face a new problem; individuals with SMID often exhibit additional physical problems in adulthood, which their improved life expectancy may reveal. For example, obesity is associated with high morbidity and mortality in adults with CP due to an increased risk of cardiometabolic disease [10]. With increasing age, eating and swallowing functions may also be impaired in adults with CP [11]. Additionally, those who take certain medications (especially for epilepsy) or have inadequate nutrition are at risk of developing osteoporosis and fragility fractures [10,11].

Furthermore, it is often difficult to manage kidney/urinary complications in clinical practice, especially kidney/urinary stones, whether in children or adults with SMID, as there are few reports on adequate management [12]. Generally, most patients with kidney stones remain asymptomatic for a long period; however, the incidence of stone-related episodes, such as pain, urinary tract infection (UTI), or urinary tract obstruction, increases with disease duration [13]. Moreover, recent evidence has shown a consistent relationship between a history of nephrolithiasis and an increased risk of chronic kidney disease (CKD) and end-stage renal disease [14,15]. Most people with SMID cannot express symptoms associated with kidney/urinary stones, making it difficult to detect and treat at an early stage. Therefore, patients with SMID are presumed to have a greater risk of urinary tract obstruction due to increased stone growth, subsequent hydronephrosis, and CKD. The potential impact of kidney infections and UTIs on a patient’s general health is enormous; however, evidence of their incidence and health burden is scarce.

This study investigated the longitudinal incidence of kidney/urinary stones in individuals with SMID and explored their health burden.

## Materials and methods

This study was approved by the National Center of Neurology and Psychiatry ethics committee on July 7, 2021 (examination number 2021-422) and conformed to the provisions of the Declaration of Helsinki. This article was previously posted to the SSRN preprint server on February 29, 2024.

Patient identification

This was a retrospective, case-control study conducted in the two wards for the long-term hospitalization of patients with SMID at our hospital. We identified patients who met all the following criteria: a) hospitalization in the wards for >10 years by June 2021; b) two or more assessments by abdominal ultrasonography or computed tomography (CT); and c) absence of kidney or urinary stones on the first imaging examination.

We excluded patients with neuromuscular diseases, primary illnesses strongly associated with future renal dysfunction (e.g., tuberous sclerosis complex, Menkes disease), and congenital malformations of the kidneys and urinary tracts. Each patient was retrospectively followed up from the first imaging examination until June 2021 or the date of death, which we defined as the observation period.

Clinical information

We reviewed the medical records of the included patients, obtaining clinical information, including sex, age, underlying condition, motor and eating function at inclusion, medication during the observation period, imaging examinations (abdominal CT or ultrasonography), and health burden events. Underlying conditions were categorized according to the clinical diagnosis made by the attending physician. We classified patients’ motor function into “bedridden,” “rolling,” and “sitting,” and eating function into “oral feeding” and “tube feeding (via nasogastric tube or gastrostomy).” UTI was clinically diagnosed when a patient exhibited symptoms (including fever and/or tachycardia) and laboratory findings (including leukocyturia) and benefited from antimicrobial treatment; positive urine cultures were not mandatory. Recurrent UTI was defined as developing two or more UTIs during the observation period. Attending physicians routinely, or at the time of need, examined the cystatin C levels in blood tests as an indicator of renal function. Serum cystatin C is considered an early and accurate biomarker of CKD [16]; therefore, we defined renal dysfunction as repeated elevation of cystatin C above the normal range (0.53-0.95 mg/L).

Main outcome measure: cumulative incidence of kidney/urinary stone formation

We checked whether kidney/urinary stones were present at each imaging examination. The cumulative incidence rate of kidney/urinary stones was calculated, and we set the five- and 10-year cumulative incidence rates as the main outcome measures. We classified patients as having kidney/urinary stones if they met either of the following criteria: a) ultrasonography showing strong echo with acoustic shadows; and b) abdominal CT with an obvious hyperdense lesion, determined to be a kidney/urinary stone by radiologists. We did not include renal calcification as a kidney stone on ultrasonography. Abdominal CT/ultrasonography was performed at the attending physician’s discretion.

Secondary outcome measures: health burden events and possible risk factors related to kidney/urinary stone formation

To explore whether kidney/urinary stone formation influenced the health conditions of patients with SMID, we investigated the occurrence of health burden events, including recurrent UTI, hydronephrosis, renal dysfunction, and death. We compared the incidence of these events between patients who developed kidney/urinary stones and those who did not. We also determined the cause of death during the observation period.

Additionally, we explored the possible risk factors for stone formation by comparing the clinical characteristics of patients with and without stone formation.

Statistical analysis

The cumulative incidence of urinary stones was analyzed using the Kaplan-Meier method. We used Fisher’s exact test to compare the incidence of health burden events in patients with and without stone formation. To compare the clinical characteristics of patients with and without stone formation, we conducted Fisher’s exact test for categorical variables and Welch’s t-test for continuous variables. P < 0.05 was considered statistically significant.

All statistical analyses were conducted using EZR software (Saitama Medical Center, Jichi Medical University, Saitama, Japan), a graphical user interface for R (The R Foundation for Statistical Computing, Vienna, Austria) [17].

## Results

Patients and clinical information

**Figure 1:**
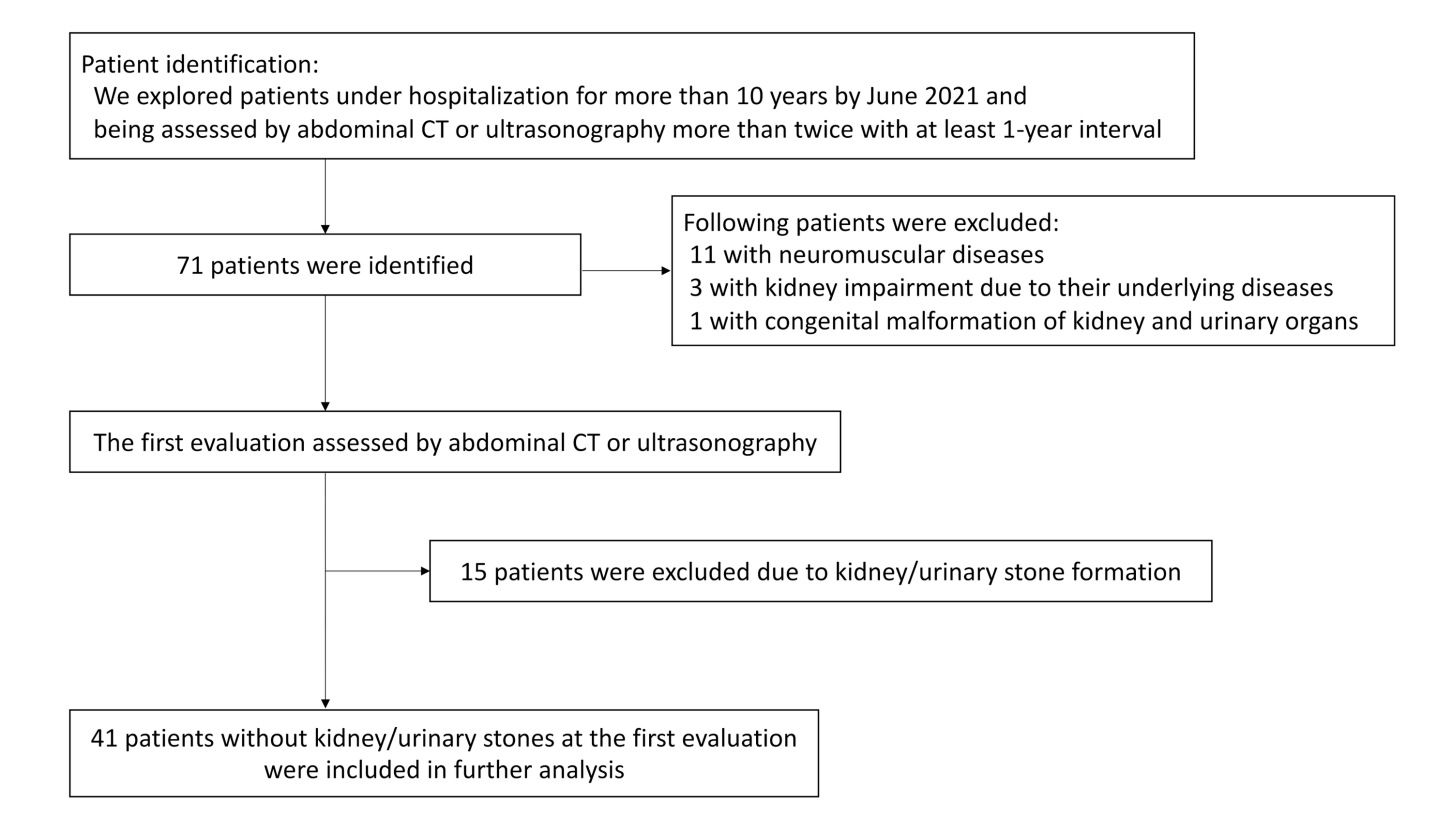
Flow diagram demonstrating inclusion and exclusion criteria Abbreviations: CT, computed tomography.

We identified 41 patients who met the inclusion criteria (19 men and 22 women, median age of 28 at the first evaluation; Figure 1). Stone formation was detected in 11 patients (27%; eight men and three women) during the observation period. Among them, kidney stones were confirmed in seven patients, ureteral stones in 0, and both in four. None of the patients with stone formation exhibited symptoms that imply the occurrence of stone attacks. The age at the time of stone confirmation was < 20 years in two patients; three patients were in their 30s, five were in their 40s, and one was in their 50s.

The clinical characteristics of the patients are summarized in Table 1. CP was the most common underlying condition, accounting for more than half of patients with or without stone formation (55 vs. 57%). In total, 27 (66%) patients were classified as bedridden, 29 (71%) as tube-feeding, and 24 (59%) were taking medications that were considered risk factors for stone formation (with an overlap), of which nine patients (22%) were treated with zonisamide, and 17 (41%) were receiving active vitamin D preparation.

**Table 1:** Clinical characteristics of the patients* *No significant differences for any items listed between the two groups. **Those identified in the neonatal period are considered cerebral palsy. Abbreviations: CT, computed tomography; US, ultrasound.

	Total (n=41)	Stone formation + (n=11)	Stone formation - (n=30)
Sex (male/female)	19 / 22	8 / 3	11 / 19
Age at start of observation (years, median (range))	28 (8-50)	27 (9-50)	29.5 (8-47)
Observation length (years, median (range))	8 (2-20)	8 (2-19)	8.5 (5-20)
Number of CT/US examinations (median (times))	9 (3-18)	11 (6-18)	9 (3-18)
Underlying condition			
Cerebral palsy (%)	23 (56)	6 (55)	17 (57)
Congenital metabolic disorders (%)	4 (10)	1 (9)	3 (10)
Degenerative neurological disorder (%)	4 (10)	1 (9)	3 (10)
Chromosomal abnormality (%)	2 (5)	0 (0)	2 (7)
Hypoxic ischemic encephalopathy (%)**	2 (5)	1 (9)	1 (3)
Subacute sclerosing panencephalitis (%)	2 (5)	2 (18)	0 (0)
Septo-optic dysplasia (%)	2 (5)	0 (0)	2 (7)
Unclassified (%)	2 (5)	0 (0)	2 (7)
Motor function at inclusion			
Bedridden (%)	27 (66)	9 (82)	18 (60)
Rolling (%)	5 (12)	1 (9)	4 (13)
Sitting (%)	9 (22)	1 (9)	8 (27)
Tube feeding at inclusion (%)	29 (71)	7 (64)	19 (63)
Drugs considered as risk factors for stone formation (%)	24 (59)	7 (64)	17 (57)
Zonisamide (%)	9 (22)	2 (18)	7 (23)
Topiramate (%)	1 (2)	0 (0)	1 (3)
Acetazolamide (%)	1 (2)	0 (0)	1 (3)
Calcium preparation (%)	5 (12)	1 (9)	4 (13)
Active vitamin D preparation (%)	17 (41)	7 (64)	10 (33)

Primary outcome: cumulative incidence of kidney/urinary stone formation

**Figure 2:**
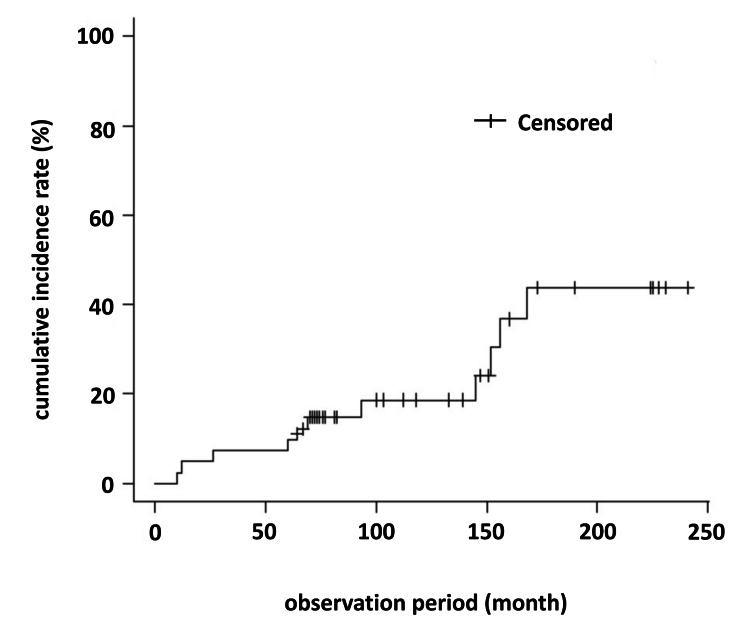
Kaplan Meier curve of the cumulative incidence rate for stone formation At five years of observation, the cumulative incidence rate was 9.8% (95% confidence interval (CI), 3.8–23.9). At 10 years of observation, the cumulative incidence rate was 18.7% (95% CI, 9.2–35.7).

The Kaplan-Meier curve of the cumulative incidence rate of stones is displayed in Figure 2. The observation period ranged from 10 to 241 months, with a median of 100 months. Including the first evaluation, we performed abdominal imaging examinations (ultrasonography or CT) three to 18 times (median, 11) per patient during the observation period. The cumulative incidence rate of stone formation at five- and 10-years was 9.8% (95% confidence interval (CI), 3.8-23.9) and 18.7% (95% CI, 9.2-35.7), respectively.

Secondary outcome: health burden events and possible risk factors related to kidney/urinary stone formation

We compared health burden events, including recurrent UTI, hydronephrosis, renal dysfunction, and death, among patients with or without stone formation (Table 2). No significant differences were observed in the occurrence of hydronephrosis or renal dysfunction; however, recurrent UTI and death were significantly more frequent in patients with stone formation. In particular, six (55%) patients in the stone formation group and two (7%) in the non-stone formation group died during the observation period. The clinical characteristics of patients who died are presented in Table 3. In the stone formation group, the causes of death were respiratory diseases (including pneumonia and respiratory failure) in three patients, UTI and subsequent septic shock in one, carcinomatous peritonitis in one, and unknown causes (sudden death) in one. In the non-stone formation group, the causes of death were acute pancreatitis in one patient and multiorgan failure in one. All patients in both groups were “bedridden” in motor function and “tube feeding” in eating function. Only one patient was classified as having CP as an underlying condition among the patients who died. 

**Table 2:** Health burden events that occurred in patients with/without stone formation

	Total (n = 41)	Stone formation + (n = 11)	Stone formation - (n = 30)	p value
Recurrent urinary tract infection (%)	6 (15)	4 (36)	2 (7)	0.04
Hydronephrosis (%)	9 (22)	3 (27)	6 (20)	0.7
Renal dysfunction (%)	22 (54)	7 (64)	15 (50)	0.5
Death (%)	9 (22)	6 (55)	2 (7)	0.002

**Table 3:** Clinical characteristics of the patients who died Abbreviations: AHC, alternating hemiplegia of childhood; DRPLA, dentatorubral-pallidoluysian atrophy; HIE, hypoxic-ischemic encephalopathy; SSPE, subacute sclerosing panencephalitis; UTI, urinary tract infection.

Case	1	2	3	4	5	6	7	8
Stone formation	+	+	+	+	+	+	-	-
Sex (male = M, female = F)	M	F	F	M	M	M	F	F
Age of death (years)	18	34	41	41	42	43	15	29
Cause of death	Unknown (cardiac arrest)	Respiratory failure	Pneumonia	Colon cancer, carcinomatous peritonitis	Acute pancreatitis, Respiratory failure	UTI, Septic shock	Acute pancreatitis	Multi-organ failure
Underlying condition	Cerebral palsy	Krabbe disease	AHC	HIE	SSPE	SSPE	DRPLA	Cryptophthalmos syndrome
Motor function	Bedridden	Bedridden	Bedridden	Bedridden	Bedridden	Bedridden	Bedridden	Bedridden
Tube feeding	+	+	+	+	+	+	+	+
Recurrent urinary tract infection	+	-	-	-	+	+	-	-
Hydronephrosis	-	-	-	-	+	+	-	+
Renal dysfunction	+	+	+	-	+	+	+	+

We compared the clinical characteristics in Table 1 between patients in the stone formation group and those in the non-stone formation group and found no significant differences for any items.

## Discussion

To our knowledge, this is the first study to report the longitudinal incidence of kidney and urinary stones in patients with SMID. The cumulative incidence of stones over five and 10 years was approximately 10% and 20%, respectively. We also found that patients who developed kidney/urinary stone formation died more frequently than those without stone formation. Although it is widely accepted that immobility can facilitate kidney/urinary stone formation [18,19], surprisingly, in our best literature search, we failed to find the longitudinal incidence of urinary stones in bedridden persons, if not limited to persons with SMID. Adults with SMID are presumed to have limited access to healthcare services [20], which makes it difficult for them to obtain regular medical tests. Indeed, our cohort is a small and highly biased population, but because of this, we could analyze their time-course change. We believe our findings will help improve the daily care of patients with SMID, contributing to their long-term kidney health.

In this study, the incidence of kidney/urinary stones in individuals with SMID was higher than that in the general population. For example, the annual incidence rate of first-episode upper urinary tract stones in Japan was estimated to be 0.14% (0.19% in men and 0.09% in women) in 2015 [21]. The lifetime incidence rates were 15.1% in men (1 in 7) and 6.8% in women (1 in 15) in 2005 [22]. Indeed, the comparison should be interpreted cautiously, as the definition of positive kidney/urinary stones might differ. Our cohort’s cumulative incidence rate over 10 years was higher than the lifetime incidence rate of the general population. The peak age of the first kidney/urinary stones episode in 2015 was the 50s in men and 60s in women [21,23]. In our study, the highest incidence of kidney/urinary stones was between the ages of 40 and 49 years; however, five (45%) patients younger than 40 years of age had kidney/urinary stones, which may be earlier than that in the general population. Therefore, clinicians caring for patients with SMID should be aware that these patients may develop kidney/urinary stones at a younger age. As most individuals with SMID have difficulty conveying their symptoms to their caregivers, regular assessment using abdominal imaging tests is recommended. 

We found that six of the 11 patients in the stone formation group died during the observation period; however, it is important to highlight that the health burden of stone formation on persons with SMID could not be determined. The direct causes of death varied, with most considered unrelated to the kidney/urinary stones. We speculated that the formation of stones itself did not trigger fatal events; however, it might reflect the poorer general condition of the patients. As many underlying conditions of the deceased patients with kidney/urinary stones were progressive neurodegenerative diseases (including subacute sclerosing panencephalitis and dentatorubral-pallidoluysian atrophy), their motor function might be much more severely impaired than “average” bedridden patients. Although promoting a sitting position if possible may be useful for preventing stone formation, it may be difficult for most persons with SMID, as they often exhibit hip joint problems including contracture and dislocation [24]. In future studies, we aim to investigate the efficacy of prophylactical medication and aggressive physiotherapy in preventing stone formation and the prophylactic removal of kidney/urinary stones in decreasing future mortality risk in persons with SMID. 

Several risk factors for stone formation in patients with SMID have been reported in Japan. For instance, Mano et al. reported oral intake function and active vitamin D preparations, and Ishikawa et al. reported that topiramate, an antiepileptic drug, is associated with stone formation [25,26]. We also explored previously reported factors related to stone formation. Some patients in the stone formation group took medications such as vitamin D preparations or zonisamide; however, we could not find a significant association between these factors and stone formation in our cohort. We believe that there are several reasons for this: patients in our cohort were managed similarly for long periods, and we did not consider the duration and dosage of their medications, including antiepileptic drugs.

This study had some limitations. First, this was a retrospective study. Second, statistical power was limited owing to a small sample size. More extensive studies are desired to establish the significance of our findings. Third, the underlying conditions were determined solely by clinical diagnosis. In particular, CP is not based on insults during the perinatal or neonatal periods. For example, patients with microdeletion syndromes may be misclassified as having CP. Fourth, the patient’s timing, frequency, and type of ultrasonography or CT varied. Fifth, we could not evaluate the temporal relationship between the timing of some health burden events (such as recurrent UTI and hydronephrosis) and stone formation. Therefore, we cannot discuss the clinical importance of recurrent UTIs and whether recurrent UTIs facilitate stone formation.

## Conclusions

We are the first to describe the longitudinal incidence of renal and urinary stones in patients with SMID. The cumulative incidences of stones over the five- and 10-year periods were approximately 10% and 20%, respectively. Although a causal relationship was not suspected, our findings suggest that patients with newly developed kidney/urinary stones are associated with a higher mortality risk. Our findings highlight the importance of regular assessment with kidney/urinary imaging tests to achieve appropriate management of patients with SMID who cannot convey symptoms associated with stones.
